# Impaired Small-World Network Efficiency and Dynamic Functional Distribution in Patients with Cirrhosis

**DOI:** 10.1371/journal.pone.0035266

**Published:** 2012-05-01

**Authors:** Tun-Wei Hsu, Changwei W. Wu, Yu-Fan Cheng, Hsiu-Ling Chen, Cheng-Hsien Lu, Kuan-Hung Cho, Wei-Che Lin, Ching-Po Lin

**Affiliations:** 1 Department of Biomedical Imaging and Radiological Sciences, National Yang-Ming University, Taipei, Taiwan; 2 Department of Diagnostic Radiology, Taipei Veterans General Hospital, Taipei, Taiwan; 3 Institute of Biomedical Engineering, National Central University, Taoyuan, Taiwan; 4 Department of Diagnostic Radiology, College of Medicine, Chang Gung University, Kaohsiung Chang Gung Memorial Hospital, Kaohsiung, Taiwan; 5 Department of Neurology, College of Medicine, Chang Gung University, Kaohsiung Chang Gung Memorial Hospital, Kaohsiung, Taiwan; 6 Brain Connectivity Lab, Institute of Neuroscience, National Yang-Ming University, Taipei, Taiwan; University of Cambridge, United Kingdom

## Abstract

Hepatic encephalopathy (HE) is a complex neuropsychiatric syndrome and a major complication of liver cirrhosis. Dysmetabolism of the brain, related to elevated ammonia levels, interferes with intercortical connectivity and cognitive function. For evaluation of network efficiency, a ‘small-world’ network model can quantify the effectiveness of information transfer within brain networks. This study aimed to use small-world topology to investigate abnormalities of neuronal connectivity among widely distributed brain regions in patients with liver cirrhosis using resting-state functional magnetic resonance imaging (rs-fMRI). Seventeen cirrhotic patients without HE, 9 with minimal HE, 9 with overt HE, and 35 healthy controls were compared. The interregional correlation matrix was obtained by averaging the rs-fMRI time series over all voxels in each of the 90 regions using the automated anatomical labeling model. Cost and correlation threshold values were then applied to construct the functional brain network. The absolute and relative network efficiencies were calculated; quantifying distinct aspects of the local and global topological network organization. Correlations between network topology parameters, ammonia levels, and the severity of HE were determined using linear regression and ANOVA. The local and global topological efficiencies of the functional connectivity network were significantly disrupted in HE patients; showing abnormal small-world properties. Alterations in regional characteristics, including nodal efficiency and nodal strength, occurred predominantly in the association, primary, and limbic/paralimbic regions. The degree of network organization disruption depended on the severity of HE. Ammonia levels were also significantly associated with the alterations in local network properties. Results indicated that alterations in the rs-fMRI network topology of the brain were associated with HE grade; and that focal or diffuse lesions disturbed the functional network to further alter the global topology and efficiency of the whole brain network. These findings provide insights into the functional changes in the human brain in HE.

## Introduction

Liver cirrhosis is frequently associated with a wide range of neuropsychiatric abnormalities including personality disorders and inappropriate affective, behavioral, and sleep disturbances. Patients with acute liver failure can succumb to neurologic death, with brain edema and intracranial hypertension [1]. This syndrome is termed hepatic encephalopathy (HE); and graded according to 4 stages of severity on the basis of clinical examination [2], [3]. Increased cerebral ammonia uptake, impaired metabolism, and decreased glucose utilization occur in several brain regions in patients with liver cirrhosis; and undergo significant alterations depending on the severity of HE [4]. Although it is not yet well understood, investigators have extensively investigated the pathophysiology of HE with the aim of developing effective therapies to prevent its onset.

The results from some studies have suggested that patients with HE might have disturbed brain energy metabolism and intracranial hemodynamics [5]–[7]. In early stages of HE, the mean dominant frequency and spectral electroencephalography (EEG) analysis can predict overt HE [5]. However, EEG studies only cortical activity, which reduces its concordance with subcortical components [6]. Evoked potentials are the latency between application of a stimulus and the brain’s ability to sense it; and represent one subclass of event-related potentials. In a study on event-related potentials in patients with cirrhosis, Schiff et al observed dysfunctional connectivity due to decline in extrastriate cortex top-down processes with preservation of bottom-up processes [8]. Evoked potentials are insensitive to changes in HE and require active patient cooperation for their assessment. These are, thus, useful only for the detection of early HE stages [9].

Neuroimaging studies in cirrhotic patients have described early impairment of the neural connectivity mechanism and abnormal coupling between visual judgment areas in HE [10]. The disruptions in interregional brain connectivity lead to the failure of functional integration within the brain. This may partially account for the deficits in cognition and behavior in these patients with cirrhosis. Further to these findings, little is known concerning the alterations in the global/local intracranial functional network in chronic liver cirrhosis and their relationship to disease severity.

Several studies have demonstrated that the human brain is organized intrinsically as highly modular small-world architectures capable of efficiently transferring information at a low wiring cost, and forming highly connected hub regions [11]–[18]. Functional segregation and integration are two major organizational principles [19]. Normal brain performance usually requires a balance between local specialization and global integration of brain functional connectivity. The small-world topology of the human brain supports both segregated and integrated information processing. It has been attributed to the brain’s network organization; allowing the brain to be more resilient to pathological attack [15], [20], [21], with minimal wiring costs. Previous investigations have observed alterations in small-world topology in patients with declining states of consciousness [22], Alzheimer’s disease [23], [24], and cognitive disorders related to aging [11], [25]. In the present study, the supposition was that HE patients might also suffer from alterations in small-world topology which related to extensions of consciousness and cognitive impairment.

To investigate this hypothesis, patients with cirrhosis with 3 grades of encephalopathy (no HE, minimal HE, and overt HE) were enrolled for resting-state fMRI (rs-fMRI) analysis. The advantage of rs-fMRI is that it avoids the need for training and differential performances on cognitive tasks, and also allows the quantification of functional connectivity (FC) by measuring the correlations or interdependencies between intrinsic blood oxygenation level dependent (BOLD) signal fluctuations of distributed brain areas. Resting-state fMRI data were obtained to investigate the differences in the organizational patterns of functional networks between patients with differing grades of HE. Results suggested dissimilar modifications to the brain organization patterns, depending on focal insults or global alterations caused by the severity of HE. The affected global and local economic performances of functional brain networks in HE might be associated with the accumulation of ammonia.

## Materials and Methods

### Participants

From August 2009 to December 2010, patients with cirrhosis and awaiting liver transplantation were enrolled from the Surgery Outpatient Clinic of Chang Gung Memorial Hospital, Kaohsiung Medical Center. Thirty-five patients (26 men and 9 women; mean age, 54.2 years; range, 34 to 68 years) were recruited and received both neuroimaging and neuropsychological (NP) examinations. The hospital’s Institutional Review Committee on Human Research approved the study.

Patients with liver cirrhosis were excluded if they had any history of drug abuse, psychiatric or neurological illness, or head injury. Cirrhosis was diagnosed according to clinical and imaging features [26]. The Child-Pugh score was applied for functional status evaluation [27]. Eighteen patients were classified as Child-Pugh class B and 17 as Child-Pugh class C. Overt HE (OHE) was graded by the West Haven criteria [28]. Patients with grade IV OHE and those requiring sedation for MRI were excluded from the study. All patients underwent laboratory screening, including albumin, creatinine, bilirubin, prothrombin time, international normalized ratio, aspartate aminotransferase, and serum venous ammonia levels, on the same day as the MRI scans and NP tests.

For comparison, 35 healthy volunteers (22 men and 13 women; median age, 51 years; range, 26 to 67 years), without any medical history of neurological disease, were recruited through advertising within the hospital and served as the control group. All volunteers received detailed clinical and neurological examinations on the same day of the MRI scans.

**Table 1:** Cortical and sub-cortical regions defined in Automated Anatomical Labeling template image in standard stereotaxic space.

Regions name	Abbreviation	Classification	Regions name	Abbreviation	Classification
Superior frontal gyrus, dorsalateral	SFGdor	Association	Superior parietal gyrus	SPG	Association
Superior frontal gyrus, orbital	SFGorb	Paralimbic	Paracentral lobule	PCL	Association
Superior frontal gyrus, medial	SFGmed	Association	Postcentral gyrus	PoCG	Primary
Superior frontal gyrus, medial orbital	SFGmorb	Paralimbic	Inferior parietal gyrus	IPG	Association
Middle frontal gyrus	MFG	Association	Supramarginal gyrus	SMG	Association
Middle frontal gyrus, orbital	MFGorb	Paralimbic	Angular gyrus	ANG	Association
Inferior frontal gyrus, opercular	IFGoper	Association	Precuneus	PCUN	Association
Inferior frontal gyrus, triangular	IFGtri	Association	Posterior cingulate gyrus	PCC	Association
Inferior frontal gyrus, orbital	IFGorb	Paralimbic			
Gyrus rectus	REG	Association	Insula	INS	Paralimbic
Anterior cingulate gyrus	ACC	Paralimbic	Thalamus	THA	Subcortical
Olfactory cortex	OLF	Paralimbic			
			Superior temporal gyrus	STG	Association
Precentral gyrus	PreCG	Primary	Superior temporal gyrus, temporal pole	STGp	Paralimbic
Supplementary motor area	SMA	Association	Middle temporal gyrus	MTG	Association
Rolandic operculum	ROL	Association	Middle temporal gyrus,temporal pole	MTGp	Paralimbic
Median- and para- Cingulate gyrus	MCC	Paralimbic	Inferior temporal gyrus	ITG	Paralimbic
			Heschl gyrus	HES	Primary
Calcarine fissure and surrounding cortex	CAL	Primary	Hippocampus	HIP	Paralimbic
Cuneus	CUN	Association	Parahippocampal gyrus	PHIP	Paralimbic
Lingual gyrus	LING	Association	Amygdala	AMG	Paralimbic
Superior occiptal gyrus	SOG	Association			
Middle occiptal gyrus	MOG	Association	Caudate nucleus	CAU	Subcortical
Inferior occiptal gyrus	IOG	Association	Lenticular nucleus, putamen	PUT	Subcortical
Fusiform gyrus	FG	Association	Lenticular nucleus, pallidum	PAL	Subcortical

**Table 2:** Demographic, clinical characteristics and neuropsychiatric test among liver cirrhosis patients and healthy control.

	Control	no HE	MHE	OHE	F or X^2^	p-value
# of subjects	35	17	9	9		
Age (years)	50.06 ± 10.23	49.95 ± 7.85	57.00 ± 5.70	60.56 ± 7.09	4.42	0.007*
Gender	23F/22M	5F/12M	1F/8M	4F/5M	1.83	0.149
Creatinine (mg/dL)	–	0.71 ± 0.35	0.74 ± 0.19	1.06 ± 0.49	0.93	0.402
GOT (IU/L)	–	66.65 ± 52.58	94.25 ± 102.07	92.00 ± 72.42	0.67	0.519
Bilirubin (mg/dL)	–	1.80 ± 0.40	3.66 ± 3.04	6.98 ± 12.43	1.91	0.162
Venous ammonia (mg/dL)	–	113.64 ± 47.75	139.10 ± 67.63	194.22 ± 81.93	3.22	0.053
Albumin (mg/dL)	–	3.35 ± 0.64	2.73 ± 0.57	2.90 ± 0.31	4.10	0.026*
International Normalized Ratio (INR)	–	1.19 ± 0.18	1.29 ± 0.28	1.60 ± 0.63	4.31	0.021*
Neuropsychiatric tests				–		
Digit-symbol	65.76 ± 22.49	59.12 ±17.46	27.22 ± 10.77	–	13.41	0.000*
Block design	35.60 ± 13.14	32.38 ± 10.65	21.80 ± 6.61	–	5.39	0.008*

Results are given as mean ± standard deviation. MHE: minimal hepatic encephalopathy; OHE, overt hepatic encephalopathy. RSPM, Raven’s Standard Progressive Matrices; CASI, Cognitive Ability Screening Instrument.

* Significant was set at p<0.05.

**Figure 1:**
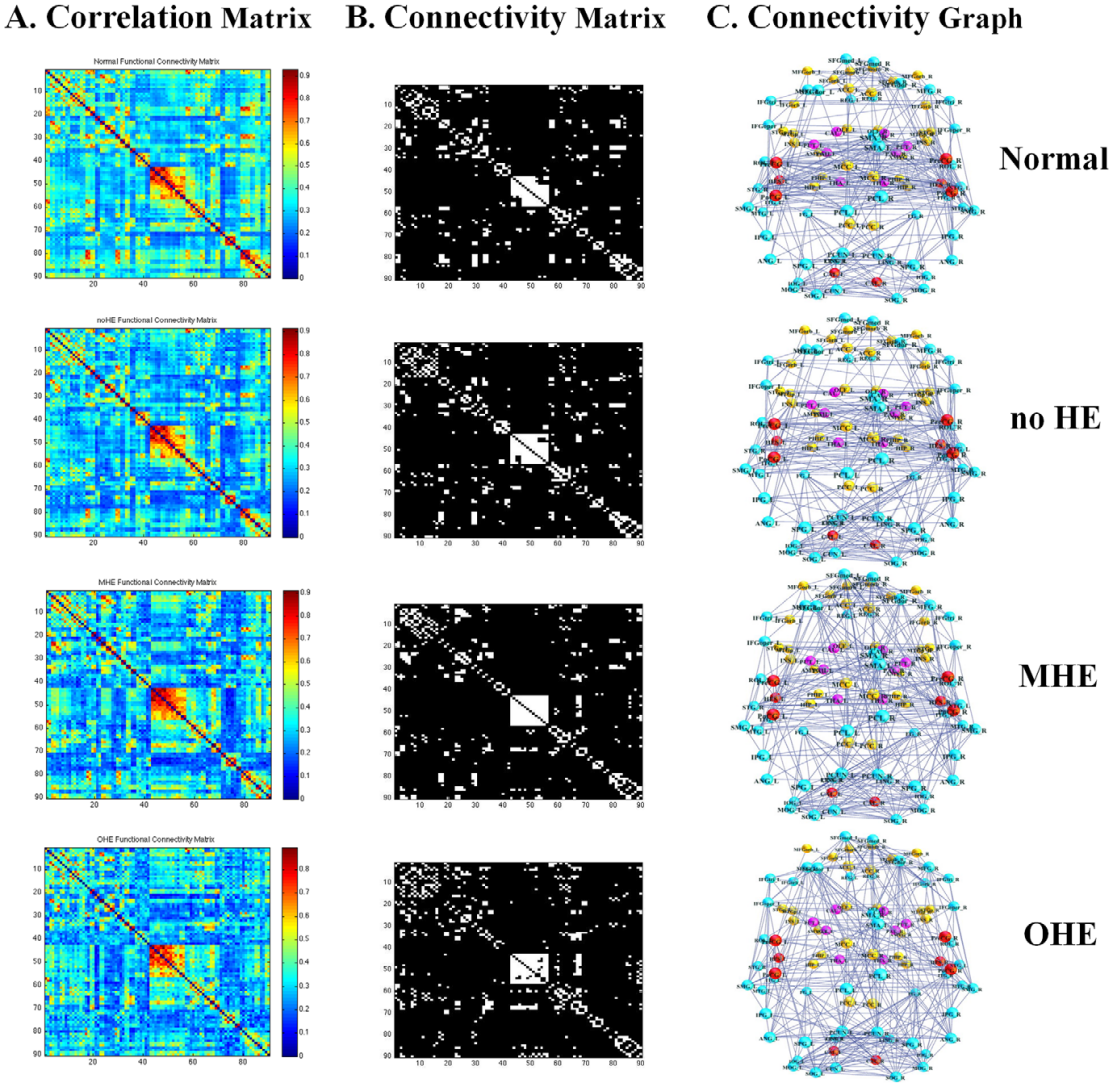
The Resting-state fMRI correlation matrices in the healthy control, no HE, MHE, and OHE groups. The A. column displays the interregional correlation matrix for each group, obtained by calculating Pearson’s correlations between the regional brain areas across subjects within the group. The color bar indicates the correlation coefficient between regions. The B column displays the binary connectivity matrices with fixed network density thresholds of 10% [59]. The C column illustrates the corresponding brain connectivity graph. The correlation matrices were further thresholded into a set of binary matrices to construct the functional networks.

**Figure 2:**
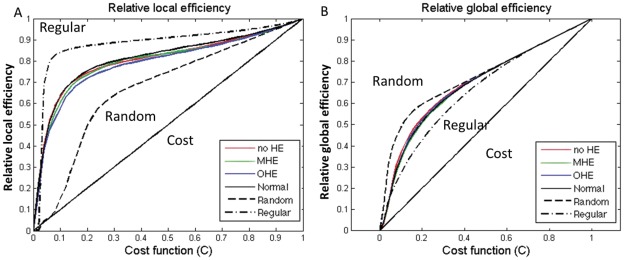
The local and global efficiency of the random, regular, and actual brain networks of each group as a function of cost. The functional brain networks showed higher local efficiency than that of the matched random networks, (A) and higher global efficiency than that of the matched regular networks (B) at a wide range of cost thresholds. Thus, the functional brain networks for each group exhibited small-world properties regardless of disease severity. The brain networks were also found to be economical since both the local and global efficiency were much higher than the required cost. Note that the regular and random networks in the plots had the same number of nodes and edges as the real networks.

**Figure 3:**
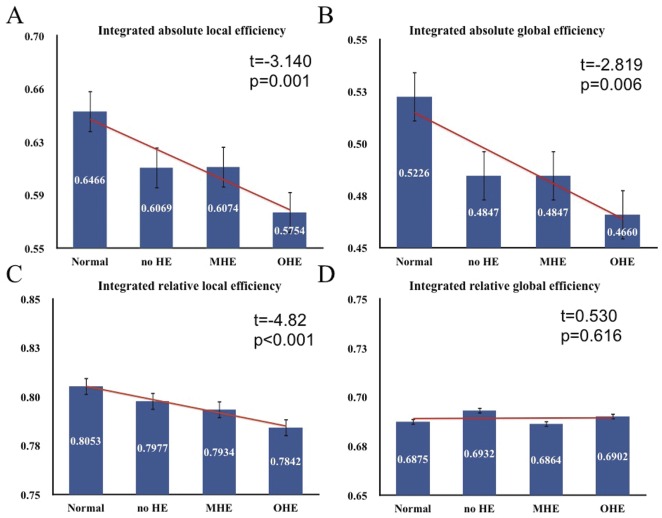
Plots showing the changes in integrated absolute and relative network efficiency. Significant decreases in absolute global and local efficiency with increasing severity of HE are observed, with non-significant increases of integrated relative global efficiency.

**Figure 4:**
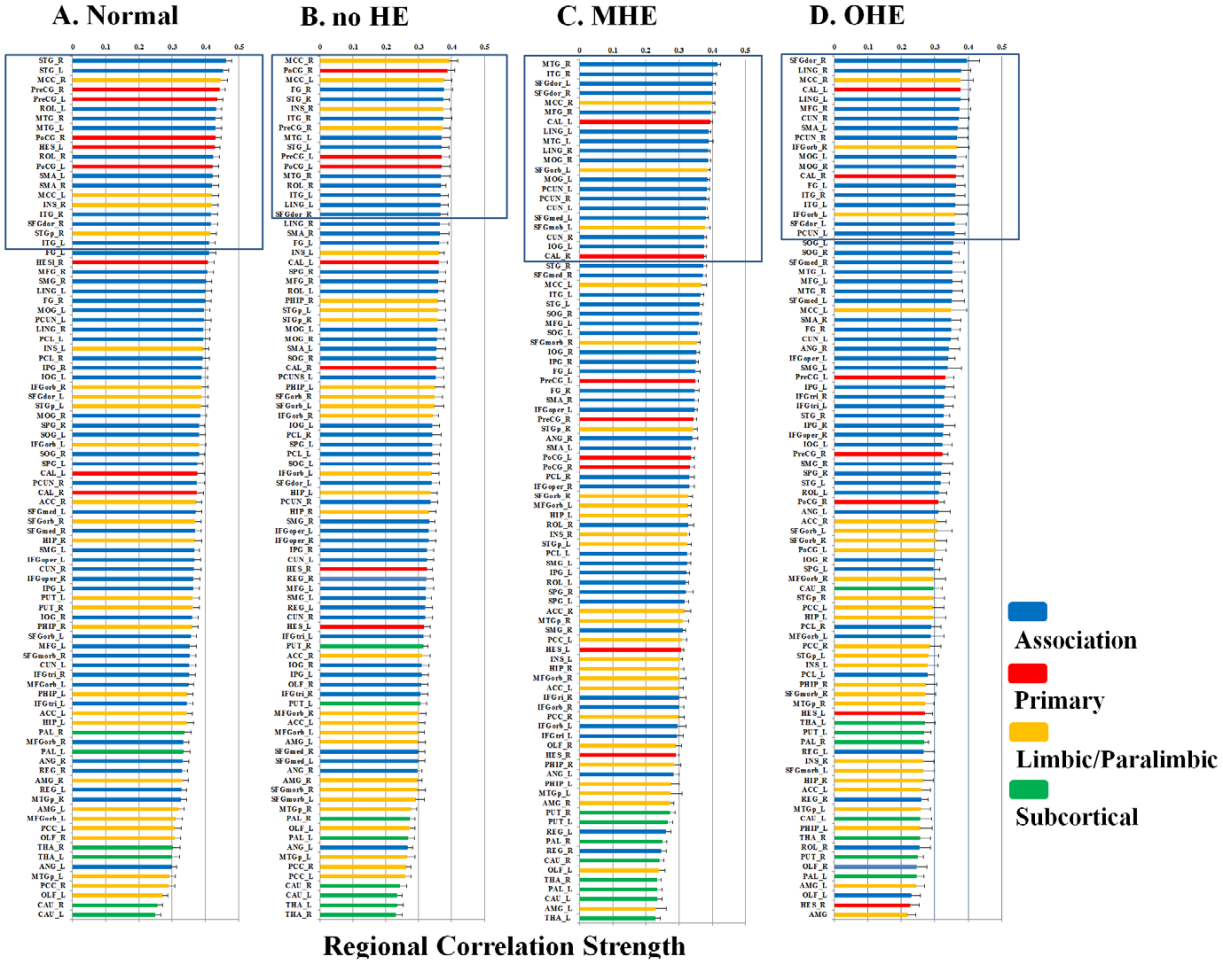
Brain regions ranked in order of decreasing regional correlation strength values in the (A) healthy control, (B) no HE, (C) MHE, and (C) OHE groups. The regional correlation strength of the nodes were redistributed and higher in healthy control compared with the HE group in the association, primary, and limbic/paralimbic cortices. The nodes shown in blue lines were 1 SD over the mean nodal connectivity strength in each group. Each box plot shows the range for the individual estimates of the regional correlation strength values in each group. The boxes are color coded to differentiate the primary sensory or motor cortex (red), heteromodal association cortex (blue), limbic or paralimbic cortex (orange), and subcortical nuclei (green).

**Figure 5:**
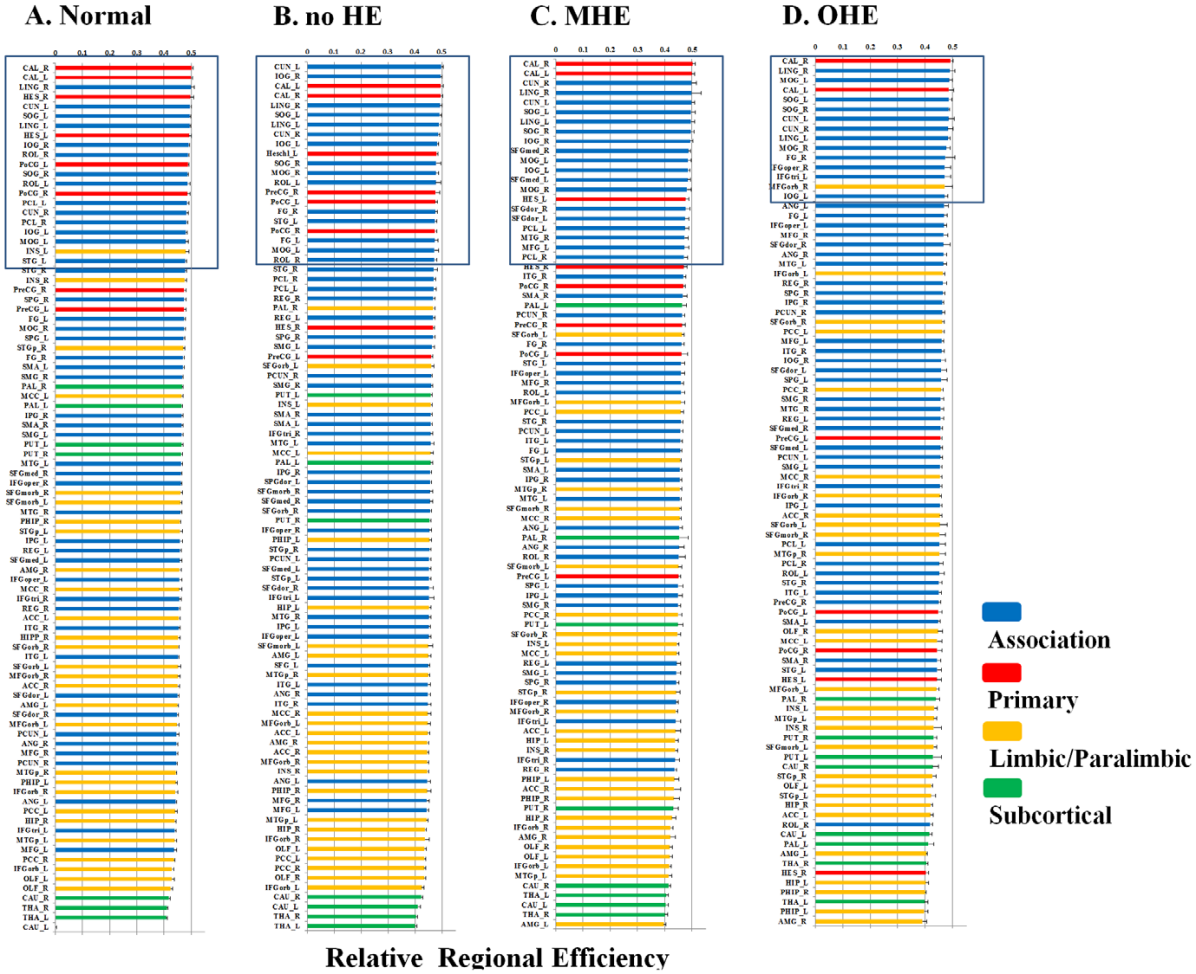
Brain regions ranked in order of decreasing regional efficiency in the (A) healthy control, (B) no HE, (C) MHE, and (C) OHE groups. The “hubs” were redistributed and the nodes that were 1 SD over the mean regional efficiency tended to be in the same regions of the association and primary cortices in each group. The HE groups had lower percentages of regional efficiency in the primary cortex for the HE (B, C, D) patients compared with the healthy controls. Each box plot shows the range for the individual estimates of the regional correlation strength values in each group. The boxes are color coded to differentiate the primary sensory or motor cortex (red), heteromodal association cortex (blue), limbic or paralimbic cortex (orange), and subcortical nuclei (green).

### Neuropsychological Tests

According to Ferenci’s report [29], minimal HE was evaluated by the Wechsler Adult Intelligence Scale III (WAIS-III) subtests, including digit-symbol and block design [30]. Abnormality in at least one of these two tests was sufficient to define a patient as having MHE. The patients with cirrhosis were divided into “no HE,” “minimal HE” (MHE), and “overt HE” (OHE) groups. Nine of the 35 patients were graded by the West Haven criteria as OHE, 9 as MHE patients with abnormal NP test results [26], [30], and 17 as no HE patients.

### MR Data Acquisition and Preprocessing

#### Data acquisition

Functional imaging data were acquired using a 3.0 T GE Signa MRI scanner (Milwaukee, WI, USA). Resting-state images, which resulted from 300 contiguous echo planar imaging whole brain functional scans (TR = 2 s, TE = 30 ms, FOV = 240 mm, flip angle 80°, matrix size 64×64, thickness = 4 mm), were collected. During the resting experiment, the scanner room was darkened and the participants were instructed to relax, with their eyes closed, without falling asleep. A 3D high-resolution T_1_-weighted anatomical image was also acquired using an inversion recovery fast spoiled gradient-recalled echo pulse sequence (TR = 9.5 ms; TE = 3.9 ms; TI = 450 ms; flip angle = 20°; field of view = 256 mm; matrix size = 512×512).

#### Resting-state fMRI preprocessing and individual analyses

Prior to preprocessing, the first 10 volumes were discarded to reach a steady-state magnetization, and to allow the participants to adapt to the scanning noise. Resting-state fMRI data preprocessing was then performed using the Statistical Parametric Mapping (SPM8, Wellcome Department of Cognitive Neurology, London, UK; http://www.fil.ion.ucl.ac.uk/spm/) and Data Processing Assistant for Resting-State fMRI (DPARSF) [31] tools. Initially, 7 patients were excluded because of head motion of more than 2.0 mm maximum displacement in any of the x, y, or z directions, or 2.5° of any angular motion throughout the course of the scan. The 35 remaining patients were divided into the no HE, MHE, and OHE groups for further data analysis. The data were also visually inspected for movement-related artifacts. The standard Montreal Neurological Institute template provided by SPM was further used for normalization with resampling to 2 mm cubic voxels and a Gaussian kernel of 6 mm (full width at half maximum) for spatial smoothing. The waveform of each voxel was finally used for removal of the linear trends of time courses; and for temporal band-pass filtering (0.01 to 0.08 Hz) to reduce low-frequency drift and high-frequency physiological noise [32], [33].

#### Nuisance signal regression

To ensure that each rs-fMRI data set was the best possible representative of spontaneous neural activities, the effects of physiological sources were minimized by regressing out estimated predictors. Nine predictors were generated including white matter (WM), cerebral spinal fluid (CSF), the global signal, and 6 motion parameters [34]. The global signal was averaged across all voxels within the brain. To generate the WM and CSF nuisance covariates, the T_1_-weighted anatomical image was segmented into gray matter, WM, and CSF maps using the SPM8 package (Wellcome Department of Cognitive Neurology). The resulting segmented WM and CSF masks were generated to ensure 80% probability of each tissue type. These individual tissue masks were then applied to the time series of each participant; the estimated predictors were calculated by averaging the time courses across all voxels within the mask.

### Network Construction

Nodes and edges are two basic elements of a network. To determine the nodes and edges of the brain networks, methods applied were similar to those described previously [15].

For each subject data set, 90 regional mean time series were estimated by averaging voxel time series within each of the 90 anatomically defined regions (Table 1) [35], which comprised the Automated Anatomical Labeling (AAL) template image [36] and served as nodes in the network construction. The interregional correlation matrix 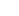 (i, j = 1, 2…N, here N = 90) was acquired in all participants by calculating Pearson’s correlation coefficients for every pair of regions which served as the edge between nodes. Two different threshold approaches were then adopted [15]. The first used the same correlation threshold values (0<R<1) which were applied to all of the group correlation matrices to construct the functional brain networks. This approach allowed the examination of the absolute network efficiency in different groups. The second approach used a cost threshold value (0<C<1), which was applied to all of the group correlation matrices. Here, the cost was calculated as the ratio of the number of actual connections divided by the maximum possible number of connections in the network. This step normalized each group network to have the same number of nodes and edges; and allowed examination of the relative network efficiency in each group.

### Network Analysis

#### Small-world efficiency

Brain functional networks have economical small-world properties which support the efficient transfer of parallel information at relatively low cost [11]. The small-world network parameters, clustering coefficient and characteristic path length, were originally proposed by Wattz and Strogatz (1998). This study employed a single network efficiency measure to quantify the FC network in healthy controls and in the patients with cirrhosis with different grades of HE. For a graph (network) G with N nodes and K edges, the global efficiency of G was calculated as [37]:
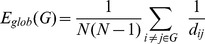
(1)In this equation,
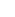
is the shortest path length between node i and j in G.The local efficiency of G was measured as [37]:
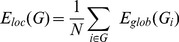
(2)Here,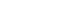 is the global efficiency of 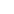, the sub-graph of the neighbors of node i. The graph G is considered to be a small-world network if it meets the following criteria:



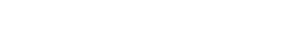
Where 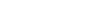, 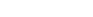, 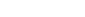, and 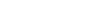 are the global and local efficiency values of node- and degree-matched regular and random networks. The integrated relative global and local efficiencies were calculated as:



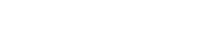
In this case, 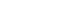
and 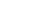 are the global and local efficiency functions of the cost variable. This process was repeated for the absolute efficiencies:



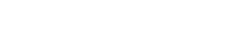



#### Regional nodal characteristics

Three measures for the nodal (regional) characteristics of functional networks in liver cirrhosis were quantified: the nodal correlation strength, its regional absolute, and the relative efficiency. The regional strength of connectivity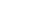 for a region i was defined as the mean of the correlations with N − 1 other regions [38]:
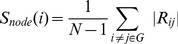
(3)In this equation, 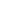 is the correlation coefficient between node i and j in G.

The regional nodal efficiency of node i was defined as the inverse of the mean harmonic shortest path length between this node and all other nodes in the network.



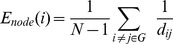
(4)This measure quantifies the importance of the nodes for communication within the network, and high regional efficiency indicates the hub roles. The integrated absolute and relative nodal efficiency was defined as
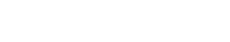
Here, 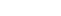 is the regional nodal efficiency function of the correlation variable; 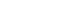 is the regional nodal efficiency function of the cost variable.

The integrated nodal efficiency enabled the characterization of nodal properties in the brain networks without the selection of a specific network threshold.

**Table 3:** Regional node characteristics versus the grade of hepatic encephalopathy by using linear regression.

		Correlation Strength		Absolute efficiency		Relative efficiency	
Anatomical regions		*t-score*	*(p-value)*	*t-score*	*(p-value)*	*t-score*	*(p-value)*
Association	Right rolandic gyrus	−4.815	(<0.001)	−5.574	(<0.001)	−5.083	(<0.001)
	Right superior temporal gyrus	−4.484	(<0.001)	−4.410	(<0.001)	−3.923	(<0.001)
	Left rolandic gyrus	−4.279	(<0.001)	−4.076	(<0.001)	−4.056	(<0.001)
	Left superior temporal gyrus	−4.202	(<0.001)	−4.440	(<0.001)	−4.247	(<0.001)
	Left paracentral lobule	−3.110	(0.003)	−3.338	(<0.001)	−2.193	(0.032)
	Right supramarginal gyrus	−3.062	(0.003)	−2.745	(0.008)	NS	
	Right gyrus rectus	−2.876	(0.005)	NS		NS	
	Right supplementary motor area	−2.815	(0.006)	−2.156	(0.035)	NS	
	Left superior parietal gyrus	−2.740	(0.016)	−2.690	(0.009)	NS	
	Right paracentral lobule	−2.632	(0.011)	−2.917	(0.005)	−2.606	(0.011)
	Left gyrus rectus	−2.381	(0.020)	NS		NS	
	Right superior paeietal gyrus	−2.226	(0.029)	−2.462	(0.016)	NS	
	Left middle temporal gyrus	−2.191	(0.032)	NS		NS	
	Right superior frontal gyrus, dorsolateral	NS		NS		2.846	(0.06)
	Left middle frontal gyrus	NS		NS		3.179	(0.002)
	Right middle frontal gyrus	NS		NS		2.610	(0.011)
	Left inferior occiptial gyrus	NS		−3.033	(0.003)	−2.137	(0.036)
	Left supplementary motor area	NS		−2.728	(0.008)	−2.554	(0.013)
Primary	Right heschl gyrus	−5.683	(<0.001)	−5.648	(<0.001)	−4.410	(<0.001)
	Left heschl gyrus	−5.464	(<0.001)	−4.697	(<0.001)	−3.680	(<0.001)
	Right precentral gyrus	−3.950	(<0.001)	−3.111	(0.003)	−2.591	(0.012)
	Right postcentral gyrus	−3.730	(<0.001)	−4.102	(<0.001)	−3.937	(<0.001)
	Left precentral gyrus	−3.235	(0.002)	−2.622	(0.011)	−2.367	(0.021)
	Left postcentral gyrus	−3.233	(0.002)	−3.803	(<0.001)	−4.014	(<0.001)
	Right calcarine fissure and surrounding cortex	NS		−2.269	(0.026)	NS	
Paralimbic	Right insula	−4.270	(<0.001)	−4.882	(<0.001)	−3.615	(<0.001)
	Left insula	−3.818	(<0.001)	−4.060	(<0.001)	−4.428	(<0.001)
	Right amygdala	−3.552	(0.001)	−4.602	(<0.001)	−3.669	(<0.001)
	Right superior temporal gyrus, temporal pole	−3.190	(0.002)	−4.414	(<0.001)	−3.956	(<0.001)
	Right hippocampus	−2.892	(0.005)	−4.024	(<0.001)	−2.871	(0.005)
	Left amygdala	−2.861	(0.006)	−3.614	(0.001)	−2.667	(0.010)
	Left superior temporal gyrus, temporal pole	−2.745	(0.008)	−3.425	(0.001)	−2.059	(0.043)
	Right parahippocampus gyrus	−2.675	(0.009)	−4.622	(<0.001)	−4.019	(<0.001)
	Left anterior cigulate gyrus	−2.598	(0.011)	−3.233	(0.002)	−2.577	(0.012)
	Left parahippocampus gyrus	−2.524	(0.014)	−3.387	(0.001)	−2.020	(0.047)
	Right anterior cigulate gyrus	−2.239	(0.028)	NS		NS	
	Left median- and para-cigulate gyrus	NS		−2.839	(0.006)	−2.228	(0.029)
	Right median- and para-cigulate gyrus	NS		−2.099	(0.040)	NS	
	Left superior frontal gyrus, medial orbital	NS		−2.655	(0.010)	NS	
	Left hippocampus	NS		−3.055	(0.003)	NS	
Subcortical	Right putamen	−3.880	(<0.001)	−4.602	(<0.001)	−3.317	(0.001)
	Left putamen	−3.441	(0.001)	−3.684	(<0.001)	−2.934	(0.005)
	Left pallidum	−3.235	(0.002)	−4.178	(<0.001)	−3.092	(0.003)
	Right pallidum	−2.749	(0.008)	−3.424	(0.001)	−2.077	(0.042)

The t-value indicates significant decreases in nodal characteristics with increasing grade hepatic encephalopathy, and vice versa. NS = non-significant. The cortical and subcortical regions were classified as primary, association, paralimbic and subcortical.

**Figure 6:**
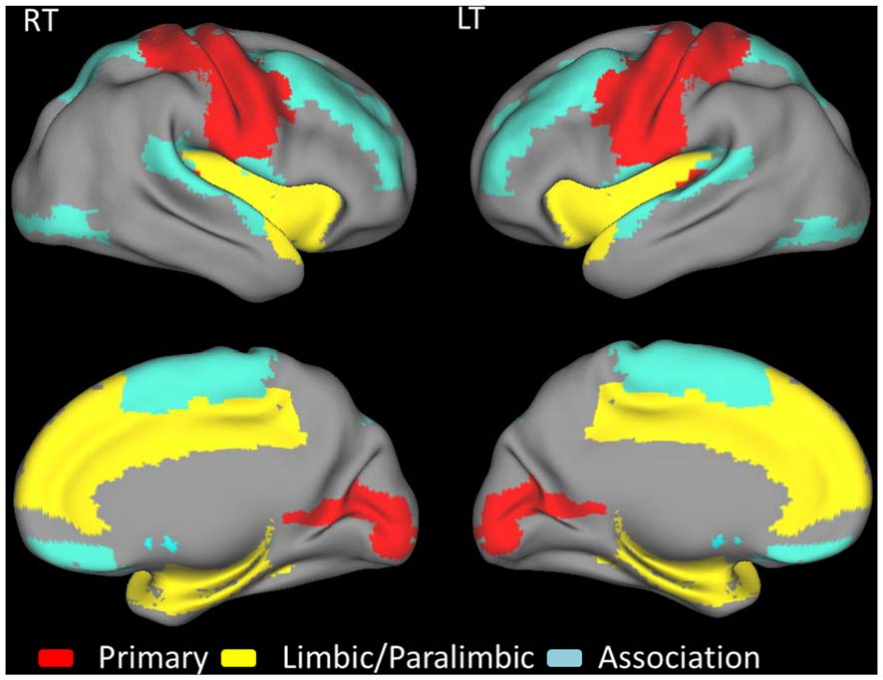
Group differences in nodal strength and relative and absolute efficiency between the healthy controls and patients with the severity of HE (Table 3). The figures show significant between group increases in the stages of HE for the left and right hemisphere.

**Figure 7:**
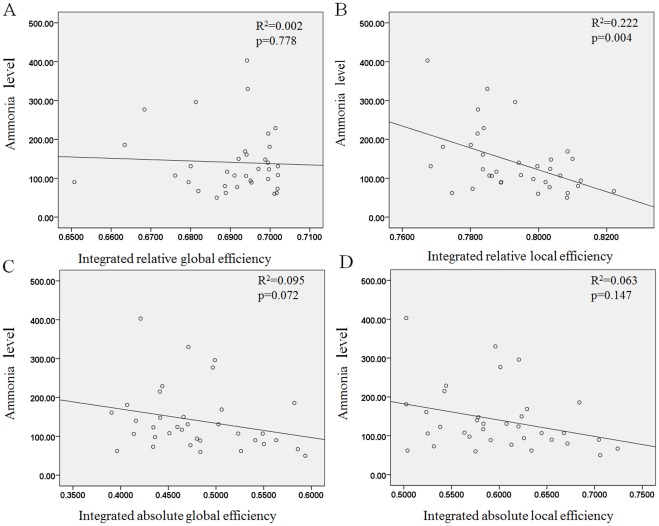
The effect of ammonia level on relative global efficiency, relative local efficiency (A and B), absolute global efficiency, and absolute local efficiency (C and D). The lower integrated relative and absolute local efficiencies that were significant and borderline significant correlated with higher ammonia levels.

**Figure 8:**
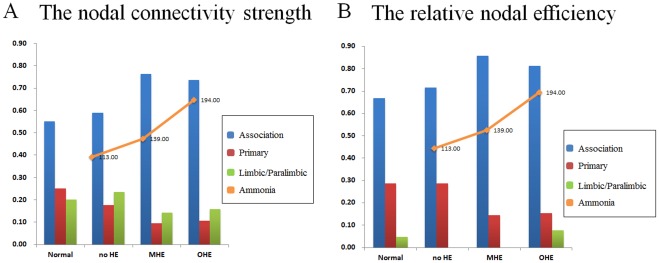
The percentages of the nodal correlation strength and efficiency distribution in the healthy controls and patient groups. Shown are the nodes of the networks, defined in terms of regional connectivity strength (A) and relative nodal efficiency (B) that were 1 SD over the mean nodal connectivity strength and the relative nodal efficiency in the healthy controls. The distribution of nodal correlation strength and efficiency in the liver cirrhotic groups showed increased weighting in the association cortex and decreased weighting in the primary cortex in the order of no HE, MHE, and OHE and correlated with the mean ammonia level. The heteromodal or unimodal association cortices (blue), primary cortices (red), limbic/paralimbic (green) and mean ammonia level (orange line) are shown.

### Statistical Analysis

Statistical analysis was performed using SPSS 13 software (SPSS Inc, Chicago, IL). The data analyses were conducted in two steps: demographic and main analyses. The demographic analyses compared the demographic variables with clinical characteristics of all subgroups of liver cirrhosis and healthy controls to identify any potentially confounding relationships. The main analyses determined the network topology between subgroups of liver cirrhosis with various degrees of severity, using linear regression and univariate ANOVA. The participants’ age and sex were applied as covariates. Network parameters, 
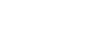
 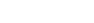, 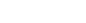, 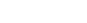, 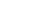, 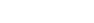, and 
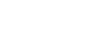
 were employed to characterize monotonic changes, with the severity of HE in the patients as a predictor. Finally, the relationship between the network efficiency and the effects of ammonia on patients was examined.

## Results

### Demographic and Clinical Characteristics

Table 2 displays the group demographic features and NP test results. Figure 1 shows the interregional functional correlation matrices, connectivity matrices, and connectivity graphs. Correlation matrices in the normal control, no HE, MHE, and OHE groups all exhibited similar patterns; with generally strong correlations between bilaterally homologous regions, and strong correlations between regions within the same lobe. Compared with the healthy controls, the overall mean strength of the absolute correlations in the patients with cirrhosis did not significantly associate with the severity of HE (F(3,66)  =  2.476, *P*>.05).

### Efficient Small-world Properties

The efficiency curves in the healthy controls and patients with cirrhosis were intermediate compared with those of the matched regular and random networks over a wide range of network costs (Figs. 2A and 2B). The local and global topological efficiencies were higher in patients with cirrhosis and healthy controls than in comparable random and regular networks [37]. These results suggested that the patients with cirrhosis and the controls both had prominent small-world properties in FC networks, consistent with previous rs-fMRI studies in healthy volunteers [11], [20], [39]. However, the relative local efficiencies (indicating the clustering coefficient) and global efficiencies (indicating the characteristic path length) of all 3 patient groups were closer to the theoretical values of random networks than those of the healthy controls. Linear regression analysis of small-world efficiency in the FC networks, with the HE severity as a predictor, showed significantly decreased small-world parameters from patients to healthy controls. Findings indicated significant decreases in the integrated absolute local efficiencies [t = −3.41, *P*<.01], integrated absolute global efficiencies [t = −2.89, *P*<.01, and integrated relative local efficiencies [t = −4.82, *P*<.001] of the FC networks (Fig. 3).

Univariate ANOVA of small-world efficiency in the FC networks also revealed significant differences in the integrated absolute (F(3,66) = 4.228, *P*<.01) and integrated relative (F(3,66) = 7.704, *P*<.001) local efficiencies. Post hoc tests with Bonferroni correction indicated that absolute and relative local efficiencies were higher in the healthy controls than in the OHE group. The absolute and relative local efficiencies in the normal?control, no HE, and MHE groups did not significantly differ.

### Regional Nodal Characteristics

In this study, the regional correlation strength of each node was a measurement of its connectivity strength to all other nodes of the cortical network; with results for all 90 brain regional nodes sorted in order of descending regional correlation strength. The primary (sensory-motor), association (heteromodal and unimodal), and limbic/paralimbic cortices had relatively higher correlation strength values in the healthy controls than in the patients with liver cirrhosis (Fig. 4). For the functional network quantifications, the 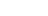 was defined as the strength of the FC network. The distribution of nodes, which had values 1 SD over the mean nodal connectivity strength 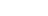 [40], differed in each group. The distributions between the association, primary, and limbic/paralimbic cortices were 55%, 25%, and 20% in the healthy controls; 59%, 18%, and 24% in the no HE group; 76%, 10%, and 14% in the MHE group; and 74%, 11%, and 16% in the OHE group, respectively.

The regional efficiency is an approach for measuring regions with highly connected nodes or hubs. High regional efficiency indicates a pivotal role in organizing network dynamics and the ability of the region to exert a strong influence on the state of additional peripheral nodes. The results for regional efficiency were similar to those for regional correlation strength (Fig. 5). The highest hubs [41] of integrated relative regional efficiency, defined as their nodal efficiency,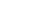, were 1 SD greater than the average of the network in the healthy controls. The nodal distributions between the association, primary, and limbic/paralimbic cortices were 67%, 29%, and 5% in the healthy controls; 71%, 29%, and 0 in the no HE group; 86%, 29%, and 0 in the MHE group; and 81%, 15%, and 8% in the OHE group, respectively.

The spatial reorganization in all of the study participants was increasingly weighted in the heteromodal or unimodal association cortices, and decreasingly weighted in the primary cortex; in the order of healthy, no HE, MHE, and OHE groups. Further comparison of the regional node characteristics, using a linear regression method of analysis, revealed that increased severity of HE was associated with significantly reduced node strength and integrated relative and absolute regional efficiency in the frontal and temporal cortices, including the limbic and paralimbic regions. These consist of the insula, hippocampus, parahippocampus gyrus, amygdala, temporal pole of the superior temporal gyrus, anterior and median cingulate cortex, and subcortical regions including the putamen and pallidum (Table 3 lists the details and Fig. 6 displays a network map of the brain regions with significantly reduced nodal characteristics in proportion to increased severity of HE; Table S1 shows the results of univariate ANOVA of the node strength and efficiency in the FC networks and post hoc tests with Bonferroni correction).

### Effects of Ammonia on Network Efficiency

Low integrated relative efficiency was the only factor to significantly correlate with high ammonia levels (*P*<.01, R^2^  =  0.22) in the patients with liver cirrhosis. These patients displayed a trend for hyperammonemia and decreased absolute, relative local and absolute global efficiencies; although this trend was not statistically significant (Fig. 7). In all 3 cirrhotic groups, hyperammonemia was also associated with increased nodal weighting in the heteromodal or unimodal association cortices, and decreased weighting in the primary cortex in the regions with the highest nodal correlation strength and relative regional efficiency (Fig. 8).

## Discussion

### Summary of the Results

(1) Disruption to the local and global topological organization of the FC network in patients with HE; with abnormal small-world properties and topological efficiency, especially in the OHE group; (2) alterations in the regional characteristics, including nodal efficiency and nodal strength, in patients with HE; predominantly in the numbers of the heteromodal or unimodal association, primary, and limbic/paralimbic regions; (3) that ammonia levels in patients with liver cirrhosis were associated with the alterations in local network properties. These findings supported the hypothesis that HE is characterized by the loss of small-world network characteristics.

### Small-world Properties of Functional Brain Networks in Patients with Liver Cirrhosis

A suitable balance between local specialization and global integration of brain functional activity [42] is required for optimal information processing. Both are believed to form the basis of many cognitive processes. In present study, the patients with HE showed lower absolute global efficiency. This indicated that information interactions between interconnected brain regions were less efficient than those of the normal controls. This was supported by the results for global efficiency (an index of functional integration), which is mainly associated with long range connections ensuring effective interactions or rapid transfer of information between and across remote cortical regions. However, there was no significant change in the relative global efficiency in patients with liver cirrhosis. This might reflect fewer long range connections in the functional networks of these patients. It is possible that the FC in some nodes and hubs undergo reorganization, and compensate for adaptation in the relative state [15] during the process of absolute HE development.

Local efficiency (an index of functional segregation), is predominantly associated with short range connections between nearby regions which mediate modularized information processing, or fault tolerance of a network [37]. Lower absolute and relative local efficiencies in HE indicate sparse local connections of brain functional networks [43], or that some neurons are damaged and inefficient [44]. These findings suggest that the brain functional network topology in liver cirrhosis might shift toward a pattern with increasing random configurations with increasing severity of HE. Previous studies on brain tumors [45], Alzheimer’s disease [46], [47], epilepsy [48], and traumatic brain injury [49] have reported similar phenomena. Network randomization may, therefore, represent a final common pathway for several brain pathologies when normal connections are impaired.

Although the declined FC network in liver cirrhosis from no HE to OHE, group differences in local small-world parameters occurred only between healthy controls and the most severe HE patients. This further supports the previous clinical observations. Patients with cirrhosis and subclinical HE exhibited a “normal” appearance, unless the patient had reached the state of OHE. Local alterations in regional FC networks, therefore, precede the global changes in the no HE and MHE groups; and their remodeling may further maintain clinical performance. Regional anatomical investigation in subclinical HE might serve to increase understanding of the development of OHE, and facilitate its early diagnosis and prevention.

### Regional Nodal Characteristics in Patients with Liver Cirrhosis

In efficient absolute and relative networks, the nodes function as hubs for information processes. According to rs-fMRI findings, they exhibit a relatively high level of metabolism and receive a high blood flow supply. This might provide an explanation for these regions being the first to demonstrate involvement in HE and in degenerative processes [50]. Decreased nodal efficiency is a critical determinant of overall brain function deterioration; with subsequent impairment of high-level cognitive functions in patients with cirrhosis.

The reductions in regional correlation strength or efficiency in the patients with cirrhosis were most prominent in the heteromodal and unimodal association, primary, paralimbic, and subcortical cortices (Table 3). Nodal efficiency further declined in relation to disease severity; occurring in selective vulnerable regions including the anterior and middle cingulate gyrus, dorsolateral prefrontal cortex, orbitofrontal cortex, temporoparietal junction, supplementary motor area, and primary cortex. These results were consistent with those from previous positron emission tomographic studies which identified impaired blood flow and oxygen metabolism in the frontal cortices and anterior cingulate gyrus in patients with HE [51]–[53]. Most of these defects are associated with the attention network in executive abilities, visual memory, and visuomotor skills. The study findings are also in agreement with those from a study on subclinical encephalopathy by Trzepacz (1994).

### Effects of Ammonia on Network Efficiency

Ammonia is extremely toxic to the brain and is absorbed and metabolized by astrocytes, leading to cell swelling [54]. In previous research, hyperammonemia increased the activity of the inhibitory GABA system and decreased the energy supply to other brain cells [55]. Morphological alterations and functional modifications might cause changes in brain signal transduction, neurotransmission, synaptic plasticity, and oscillatory networks. The present study’s findings indicated a negative correlation between ammonia levels and the global and local efficiencies of the patients with liver cirrhosis, especially in the integrated relative local efficiency. This indicated that the alterations in the FC network caused by increases in ammonia concentration were related to the severity of HE.

For regional efficiency, hyperammonemia caused increased nodal weighting in the heteromodal or unimodal association cortices, and decreased weighting in the primary cortices of the HE, MHE, and OHE groups (Fig. 8). This supported the results of a study by Zafiris et al, which indicated that early impairment might occur in vulnerable primary cortices during the development of HE [10]. The increased weighting in the heteromodal or unimodal association cortices would increase the signal-to-noise ratio in the remaining connections. This might have improved the information transmission performance. Fernandez et al observed similar findings in early stage Alzheimer’s disease patients; the increased responsiveness of the association cortex reflected dynamic compensation for the impaired transmission of signals from the primary cortex [56]. The human brain might, therefore, demonstrate plasticity; leading to a proportional synaptic downscaling which leaves only the most robust connections intact, and reduces the energy requirements for the maintenance of basic brain function during the process of HE development. The heteromodal or unimodal association cortices receive convergent inputs from multiple cortical regions. These are thought to be the substrates for the maintenance of higher mental activities and consciousness in humans. Lesion, NP, and neuroimaging studies have demonstrated the significance of association cortices in specific cognitive domains [57].

### Methodological Considerations

First, the BOLD signal is an indirect measurement of neuroelectrical activity. The hemodynamic response of the BOLD signal is quite limited, due to temporal resolution, and delayed by real-time modulations of neural activity. Although rs-fMRI is an important approach for understanding the normal human brain and psychiatric diseases, some noise, such as cardiac and/or respiratory cycle-related pulsations and instrumental and thermal sources of noise, is inevitable. Head movement (rotation or translation) of the participant during scanning may also affect the stability of the rs-fMRI signals. Methods such as regression can reduce noise; although new and improved methods need development to further reduce noise from the previously mentioned sources. Second, it is not possible to eliminate the effects of heterogeneity in clinical symptoms, duration of illness, severity of symptoms, and medication on patient findings. Third, the study sample size was small (9 MHE and 9 OHE patients), due to the generally low incidence of HE. Fourth, the human brain is a complex network on multiple spatial and time scales; therefore it is difficult to select the nodes, edges, and time scale which most appropriately represent the network in the natural state of the brain. The inappropriate representation of nodes and edges in a network, and failure to consider the dynamics of the system of interest, could lead to misleading conclusions and generally poor results [58].

### Clinical Considerations

In clinical application, the rs-fMRI has advantages over task activation fMRI in that it avoids the need for training and differential performance on cognitive tasks; and also enables noninvasive measurement of human brain function in cirrhotic patients with decreased consciousness. When combined with traditional structural imaging techniques, the rs-fMRI is a powerful tool for detecting neural activation and functional connectivity in local and global brain regions in vivo. The present study’s results might contribute to future methods which facilitate the early detection, or the monitoring of progression, of HE in clinical practice.

### Conclusions

The present study’s results indicate that the resting-state network topology of the brain relates to the grade of HE. Focal or diffuse lesions might cause decreased network efficiency in patients with severe HE, as well as alterations in the whole brain functional neuronal organization. These findings further elucidate changes occurring in the functional architecture of the human brain in patients with liver cirrhosis and HE.

## Supporting Information

Table S1 **Regional node characteristics versus the grade of hepatic encephalopathy by using ANOVA and post hoc test.**
(DOC)

## References

[1] Cordoba J, Blei A (1996). Brain edema and hepatic encephalopathy.. Seminars in liver disease.

[2] Blei AT, Cordoba J (2001). Hepatic encephalopathy.. The American journal of gastroenterology.

[3] Gerber T, Schomerus H (2000). Hepatic encephalopathy in liver cirrhosis: pathogenesis, diagnosis and management.. Drugs.

[4] Lockwood AH, Weissenborn K, Bokemeyer M, Tietge U, Burchert W (2002). Correlations between cerebral glucose metabolism and neuropsychological test performance in nonalcoholic cirrhotics.. Metabolic Brain Disease.

[5] Almdal T, Schroeder T, Ranek L (1989). Cerebral blood flow and liver function in patients with encephalopathy due to acute and chronic liver diseases.. Scandinavian journal of gastroenterology.

[6] Kato A, Suzuki K, Kaneta H, Obara H, Fujishima Y (2000). Regional differences in cerebral glucose metabolism in cirrhotic patients with subclinical hepatic encephalopathy using positron emission tomography.. Hepatology research.

[7] Lockwood A, Yap E, Rhoades H (1991). Altered cerebral blood flow and glucose metabolism in patients with liver disease and minimal encephalopathy.. Journal of cerebral blood flow and metabolism.

[8] Schiff S, Mapelli D, Vallesi A, Orsato R, Gatta A (2006). Top-down and bottom-up processes in the extrastriate cortex of cirrhotic patients: an ERP study.. Clinical neurophysiology.

[9] Lai JCK, Cooper AJL (1986). Brain α-Ketoglutarate Dehydrogenase Complex: Kinetic Properties, Regional Distribution, and Effects of Inhibitors.. Journal of neurochemistry.

[10] Zafiris O, Kircheis G, Rood HA, Boers F, Haussinger D (2004). Neural mechanism underlying impaired visual judgement in the dysmetabolic brain: an fMRI study.. NeuroImage.

[11] Achard S, Bullmore E (2007). Efficiency and cost of economical brain functional networks.. PLoS Computational Biology.

[12] Chen ZJ, He Y, Rosa-Neto P, Germann J, Evans AC (2008). Revealing Modular Architecture of Human Brain Structural Networks by Using Cortical Thickness from MRI.. Cerebral Cortex.

[13] Gong G, Rosa-Neto P, Carbonell F, Chen ZJ, He Y (2009). Age- and Gender-Related Differences in the Cortical Anatomical Network.. Journal of Neuroscience.

[14] Hagmann P, Cammoun L, Gigandet X, Meuli R, Honey CJ (2008). Mapping the structural core of human cerebral cortex.. PLoS Biol.

[15] He Y, Dagher A, Chen Z, Charil A, Zijdenbos A (2009). Impaired small-world efficiency in structural cortical networks in multiple sclerosis associated with white matter lesion load.. Brain.

[16] Rubinov M, Sporns O (2010). Complex network measures of brain connectivity: Uses and interpretations.. NeuroImage.

[17] Sanabria-Diaz G, Melie-Garcia L, Iturria-Medina Y, Aleman-Gomez Y, Hernandez-Gonzalez G (2010). Surface area and cortical thickness descriptors reveal different attributes of the structural human brain networks.. NeuroImage.

[18] Vaessen MJ, Hofman PAM, Tijssen HN, Aldenkamp AP, Jansen JFA (2010). The effect and reproducibility of different clinical DTI gradient sets on small world brain connectivity measures.. NeuroImage.

[19] Sporns O, Zwi JD (2004). The small world of the cerebral cortex.. Neuroinformatics.

[20] Achard S, Salvador R, Whitcher B, Suckling J, Bullmore E (2006). A resilient, low-frequency, small-world human brain functional network with highly connected association cortical hubs.. Journal of Neuroscience.

[21] Bassett DS, Bullmore ET (2009). Human brain networks in health and disease.. Current Opinion in Neurology.

[22] Salvador R, Suckling J, Schwarzbauer C, Bullmore E (2005). Undirected graphs of frequency-dependent functional connectivity in whole brain networks.. Philosophical Transactions of the Royal Society B: Biological Sciences.

[23] He Y, Chen Z, Evans A (2008). Structural Insights into Aberrant Topological Patterns of Large-Scale Cortical Networks in Alzheimer’s Disease.. Journal of Neuroscience.

[24] Stam C, Jones B, Nolte G, Breakspear M, Scheltens P (2007). Small-world networks and functional connectivity in Alzheimer’s disease.. Cerebral Cortex.

[25] Meunier D, Achard S, Morcom A, Bullmore E (2009). Age-related changes in modular organization of human brain functional networks.. NeuroImage.

[26] Kale RA, Gupta RK, Saraswat VA, Hasan KM, Trivedi R (2006). Demonstration of interstitial cerebral edema with diffusion tensor MR imaging in type C hepatic encephalopathy.. Hepatology.

[27] Pugh R, Murray Lyon I, Dawson J, Pietroni M, Williams R (1973). Transection of the oesophagus for bleeding oesophageal varices.. British Journal of Surgery.

[28] Atterbury CE, Maddrey WC, Conn HO (1978). Neomycin-sorbitol and lactulose in the treatment of acute portal-systemic encephalopathy.. Digestive Diseases and Sciences.

[29] Ferenci P, Lockwood A, Mullen K, Tarter R, Weissenborn K (2002). Hepatic encephalopathy – definition, nomenclature, diagnosis, and quantification: final report of the working party at the 11th World Congresses of Gastroenterology, Vienna, 1998.. Hepatology.

[30] Das A, Dhiman RK, Saraswat VA, Verma M, Naik SR (2001). Prevalence and natural history of subclinical hepatic encephalopathy in cirrhosis.. Journal of Gastroenterology and Hepatology.

[31] Chao-Gan Y, Yu-Feng Z (2010). DPARSF: a MATLAB toolbox for pipeline data analysis of resting-state fMRI.. Frontiers in Systems Neuroscience.

[32] Biswal B, Yetkin F, Haughton V, Hyde J (1995). Functional connectivity in the motor cortex of resting human brain using echo-planar MRI.. Magnetic Resonance in Medicine.

[33] Lowe M, Mock B, Sorenson J (1998). Functional connectivity in single and multislice echoplanar imaging using resting-state fluctuations.. NeuroImage.

[34] Birn RM, Murphy K, Bandettini PA (2008). The effect of respiration variations on independent component analysis results of resting state functional connectivity.. Human Brain Mapping.

[35] Zwingmann C, Chatauret N, Leibfritz D, Butterworth R (2003). Selective increase of brain lactate synthesis in experimental acute liver failure: results of a [H-C] nuclear magnetic resonance study,.. Hepatology.

[36] Tzourio-Mazoyer N, Landeau B, Papathanassiou D, Crivello F, Etard O (2002). Automated anatomical labeling of activations in SPM using a macroscopic anatomical parcellation of the MNI MRI single-subject brain.. NeuroImage.

[37] Latora V, Marchiori M (2001). Efficient Behavior of Small-World Networks.. Physical Review Letters.

[38] Jiang T, He Y, Zang Y, Weng X (2004). Modulation of functional connectivity during the resting state and the motor task.. Human Brain Mapping.

[39] Wang J, Wang L, Zang Y, Yang H, Tang H (2009). Parcellation-dependent small-world brain functional networks: A resting-state fMRI study.. Human Brain Mapping.

[40] Wu K, Taki Y, Sato K, Sassa Y, Inoue K (2011). The Overlapping Community Structure of Structural Brain Network in Young Healthy Individuals.. PLoS ONE.

[41] Sporns O, Honey CJ, Kotter R (2007). Identification and classification of hubs in brain networks.. PLoS ONE.

[42] Tononi G, Edelman GM (1998). Consciousness and complexity.. Science.

[43] Micheloyannis S, Pachou E, Stam CJ, Breakspear M, Bitsios P (2006). Small-world networks and disturbed functional connectivity in schizophrenia.. Schizophrenia Research.

[44] Zhao Q, Tang Y, Feng H, Li C, Sui D (2008). The effects of neuron heterogeneity and connection mechanism in cortical networks.. Physica A: Statistical Mechanics and its Applications.

[45] Bartolomei F, Bosma I, Klein M, Baayen JC, Reijneveld JC (2006). Disturbed functional connectivity in brain tumour patients: evaluation by graph analysis of synchronization matrices.. Clinical neurophysiology.

[46] De Haan W, Pijnenburg YAL, Strijers RLM, Van Der Made Y, Van Der Flier WM (2009). Functional neural network analysis in frontotemporal dementia and Alzheimer’s disease using EEG and graph theory.. BMC neuroscience.

[47] Stam C, De Haan W, Daffertshofer A, Jones B, Manshanden I (2009). Graph theoretical analysis of magnetoencephalographic functional connectivity in Alzheimer’s disease.. Brain.

[48] Van Dellen E, Douw L, Baayen JC, Heimans JJ, Ponten SC (2009). Long-term effects of temporal lobe epilepsy on local neural networks: a graph theoretical analysis of corticography recordings.. PLoS ONE.

[49] Nakamura T, Hillary FG, Biswal BB (2009). Resting network plasticity following brain injury.. PLoS ONE.

[50] Buckner RL, Sepulcre J, Talukdar T, Krienen FM, Liu H (2009). Cortical Hubs Revealed by Intrinsic Functional Connectivity: Mapping, Assessment of Stability, and Relation to Alzheimer’s Disease.. Journal of Neuroscience.

[51] Kawatoko T, Murai K, Ibayashi S, Tsuji H, Nomiyama K (1992). Marked reduction of cerebral oxygen metabolism in patients with advanced cirrhosis: a positron emission tomography study.. Fukuoka igaku zasshi =  Hukuoka acta medica.

[52] O’Carroll R, Ebmeier K, Dougall N, Murray C, Goodwin G (1991). Regional cerebral blood flow and cognitive function in patients with chronic liver disease.. The Lancet.

[53] Trzepacz P, Tarter R, Shah A, Tringali R, Faett D (1994). SPECT scan and cognitive findings in subclinical hepatic encephalopathy.. The Journal of neuropsychiatry and clinical neurosciences.

[54] Norenberg M, Rao KVR, Jayakumar A (2005). Mechanisms of ammonia-induced astrocyte swelling.. Metabolic Brain Disease.

[55] Hertz L, Kala G (2007). Energy metabolism in brain cells: effects of elevated ammonia concentrations.. Metabolic Brain Disease.

[56] Fernandez R, Kavcic V, Duffy CJ (2007). Neurophysiologic analyses of low-and high-level visual processing in Alzheimer disease.. Neurology.

[57] Toga AW, Thompson PM (2003). Mapping brain asymmetry.. Nature Reviews Neuroscience.

[58] Butts CT (2009). Revisiting the foundations of network analysis.. Science.

[59] Greicius MD, Flores BH, Menon V, Glover GH, Solvason HB (2007). Resting-state functional connectivity in major depression: abnormally increased contributions from subgenual cingulate cortex and thalamus.. Biological psychiatry.

