# Supplementary material for: Impaired Small-World Network Efficiency and Dynamic Functional Distribution in Patients with Cirrhosis
Source: PLoS One. 2012 May 1;7(5):e35266. doi: 10.1371/journal.pone.0035266 (PMC3341390; doi:10.1371/journal.pone.0035266)
Supplement: Table S1 — Regional node characteristics versus the grade of hepatic encephalopathy by using ANOVA and post hoc test. (DOC) [file pone.0035266.s001.doc]

Table S1. Regional node characteristics versus the grade of hepatic encephalopathy by using ANOVA and post hoc test.

|  | | **Correlation Strength** | | | **Absolute efficiency** | | | **Relative efficiency** | | |
| --- | --- | --- | --- | --- | --- | --- | --- | --- | --- | --- |
| **Anatomical regions** | | ***F(3,66) (p-value)*** | | ***Bonferrnoi post hoc*** | ***F(3,66) (p-value)*** | | ***Bonferrnoi post hoc*** | ***F(3,66) (p-value)*** | | ***Bonferrnoi post hoc*** |
| ***Association*** | Right rolandic gyrus | 7.566 | <0.001 | N > OHE | 10.831 | <0.001 | (N, no HE (NS)) > OHE | 8.589 | <0.001 | (N, no HE (NS)) > (MHE,OHE (NS)) |
|  | Right superior temporal gyrus | 7.649 | <0.001 | N > (no HE, OHE(NS)) | 6.588 | 0.001 | N > OHE | 5.003 | 0.003 | N > OHE |
|  | Left rolandic gyrus | 6.703 | 0.001 | N >( MHE, OHE(NS)) | 5.407 | 0.002 | N > OHE | 5.388 | 0.002 | N > OHE |
|  | Left superior temporal gyrus | 6.542 | 0.001 | N >( no HE, OHE(NS)) | 6.411 | 0.001 | N > OHE | 6.297 | 0.001 | N > OHE |
|  | Left inferior occiptial gyrus | NS |  |  | 4.494 | 0.006 | (N, no HE (NS)) > OHE | 3.220 | 0.028 | (N, no HE (NS)) > OHE |
|  | Left paracentral lobule | NS |  |  | 3.881 | 0.013 | NS | NS |  |  |
|  | Right supramarginal gyrus | 4.092 | 0.01 | NS | 2.921 | 0.04 | NS | NS |  |  |
|  | Right gyrus rectus | 3.480 | 0.021 | NS | NS |  |  | NS |  |  |
|  | Right supplementary motor area | NS |  |  | 3.112 | 0.032 | NS | NS |  |  |
|  | Right paracentral lobule | NS |  |  | 3.088 | 0.033 | NS | NS |  |  |
|  | Left gyrus rectus | NS |  |  | NS |  |  | NS |  |  |
|  | Left superior frontal gyrus, dorsolateral | 3.283 | 0.026 | NS | 3.193 | 0.029 | NS | NS |  |  |
|  | Left middle frontal gyrus | NS |  |  | 4.440 | 0.007 | NS | NS |  |  |
|  | Right middle frontal gyrus | NS |  |  | 2.901 | 0.041 | NS | NS |  |  |
| ***Primary*** | Right heschl gyrus | 10.807 | <0.001 | N>(no HE,MHE,OHE(NS)) | 11.210 | <0.001 | (N, no HE (NS)) > OHE | 7.505 | <0.001 | (N, no HE (NS)) > OHE |
|  | Left heschl gyrus | 12.202 | <0.001 | (N,no HE (NS))>(MHE, OHE(NS)) | 7.606 | <0.001 | (N, no HE (NS)) > OHE | 4.975 | 0.004 | (N, no HE (NS)) > OHE |
|  | Right precentral gyrus | 5.487 | 0.002 | (N,no HE (NS) >(MHE, OHE(NS)) | 3.208 | 0.029 | NS | 2.281 | 0.046 | NS |
|  | Right postcentral gyrus | 4.586 | 0.006 | NS | 5.605 | 0.002 | NS | 5.301 | 0.002 | N > OHE |
|  | Left precentral gyrus | 3.829 | 0.014 | NS | 2.910 | 0.042 | NS | NS |  |  |
|  | Left postcentral gyrus | 3.442 | 0.022 | NS | 5.124 | 0.003 | NS | 5.215 | 0.003 | NS |
| ***Paralimbic*** | Right insula | 5.037 | 0.001 | N > OHE | 8.048 | <0.001 | N > (MHE,OHE(NS)) | 4.996 | 0.003 | NS |
|  | Left insula | 4.833 | 0.004 | N > OHE | 5.937 | 0.002 | N > OHE | 5.397 | 0.002 | N > OHE |
|  | Right amygdala | 4.170 | 0.009 | NS | 7.000 | <0.001 | (N, no HE (NS)) > OHE | 4.591 | 0.006 | NS |
|  | Right superior temporal gyrus, temporal pole | 3.443 | 0.022 | N > OHE | 6.894 | <0.001 | N > OHE | 5.123 | 0.003 | N>OHE |
|  | Right hippocampus | NS |  |  | 5.619 | 0.002 | N>OHE | NS |  |  |
|  | Left amygdala | 3.142 | 0.031 | NS | 4.410 | 0.007 | N>OHE | 3.123 | 0.032 | NS |
|  | Left superior temporal gyrus, temporal pole | NS |  |  | 5.06 | 0.003 | N>OHE | NS |  |  |
|  | Right parahippocampus gyrus | 2.818 | 0.046 | NS | 7.401 | <0.001 | N>OHE | 5.545 | 0.002 | (N, no HE (NS)) > OHE |
|  | Left anterior cigulate gyrus | NS |  |  | 3.884 | 0.013 | N>OHE | NS |  |  |
|  | Left parahippocampus gyrus | NS |  |  | 4.377 | 0.007 | N>OHE | NS |  |  |
|  | Right anterior cigulate gyrus | NS |  |  | NS |  |  | NS |  |  |
|  | Left median- and para-cigulate gyrus | NS |  |  | 2.917 | 0.041 | NS | NS |  |  |
|  | Right median- and para-cigulate gyrus | NS |  |  | NS |  |  | NS |  |  |
|  | Left hippocampus | NS |  |  | 3.478 | 0.021 | N>OHE | NS |  |  |
| ***Subcortical*** | Right putamen | 4.971 | 0.004 | N >OHE | 6.909 | <0.001 | N >OHE | 3.86 | 0.013 | NS |
|  | Left putamen | 4.367 | 0.007 | N >( MHE, OHE(NS)) | 4.85 | 0.004 | N >OHE | 3.04 | 0.035 | N >OHE |
|  | Left pallidum | 4.193 | 0.009 | N >MHE | 7.711 | <0.001 | (N, no HE,MHE (NS)) > OHE | 5.228 | 0.003 | (N, no HE,MHE (NS)) > OHE |
|  | Right pallidum | 3.491 | 0.02 | NS | 4.029 | 0.011 | N >OHE | NS |  |  |

The F-value indicate significant difference in nodal characteristics HE grades. NS=non-significant, N=healthy control. The cortical and subcortical regions were classify as primary, association, paralimbic and subcortical.
